# Secondary Metabolites of Halobacillus sp.: Antimicrobial and Antioxidant Activity, Biological Compatibility, and Gas Chromatography-Mass Spectrometry (GC-MS) Analysis

**DOI:** 10.7759/cureus.67246

**Published:** 2024-08-19

**Authors:** Aayisha Aathila Saleem, Gayathri Balakrishnan, Manivannan Nandhagopal

**Affiliations:** 1 Biocontrol and Microbial Products Lab, Department of Microbiology, Saveetha Medical College and Hospitals, Saveetha Institute of Medical and Technical Sciences, Saveetha University, Chennai, IND

**Keywords:** bio-compatibility, antimicrobial activity, secondary metabolites, halophilic bacteria, durg discovery

## Abstract

Background

The rise of infectious diseases and the emergence of resistant pathogens pose significant challenges to human health. In response to this global threat, researchers are exploring novel sources of bioactive compounds for effective antimicrobial therapies. One avenue of investigation is the study of halophilic bacteria and their secondary metabolites. These bacteria thrive under extreme conditions and produce valuable bioactive metabolites, which have the potential for therapeutic applications.

Methods

In this study, the potent bacterial cultures obtained from the Payanur salt pan, Tamil Nadu, were analyzed for the antimicrobial activity of their metabolites. The secondary metabolites were obtained from the halophilic bacteria by culturing the bacteria in 8% NaCl. The resultant secondary metabolites produced were extracted using ethyl acetate and their antimicrobial property was studied using the well diffusion method. The minimum inhibitory concentration (MIC) of these metabolites against five clinical pathogens, namely, *Staphylococcus aureus, Escherichia coli, Enterococcus faecalis, Pseudomonas aeruginosa, *and *Candida albicans* was determined. Their antioxidant property was studied using the DPPH (2,2-diphenyl-1-picrylhydrazyl) method and biological compatibility was determined by hemolytic assay of the secondary metabolites.

Results

The potent halophilic bacteria isolated from salt pan bacteria were phenotypically and genotypically identified as *Halobacillus *sp*.* The secondary metabolites extracted from these bacteria yielded 110 mg of crude metabolites. The antimicrobial activity of crude metabolites shows a moderate zone of inhibition of 14 mm for *P. aeruginosa*, 13 mm for *E. coli* and *C. albicans,* and 11 mm for *S. aureus*. The minimum inhibitory concentration was 128 µg/mL for *E. coli*, *P. aeruginosa,* and *C. albicans,* which was found to be the best growth inhibition concentration. The DPPH scavenging activity shows a higher activity till the concentration of 64 µg/mL. The hemolytic activity of 25% is obtained at 128 µg/mL and below 64 µg/mL, there is no hemolytic activity. The gas chromatography-mass spectrometry (GC-MS) analysis of the secondary metabolites shows the presence of 17 compounds. Among them, there were four major compounds: (i) cyclo(L-prolyl-L-valine) (probability of 95.63%), (ii) pyrrolo[1,2-a]pyrazine- 1,4-dione,hexahydro-3-(2-methylpropl) (probability of 94.45%), (iii) 2,5-piperazinedione,3,6-bis(2-methylpropyl) (probability of 71.94%) and (iv) pyrrolo[1,2-a]pyrazine-1,4-dione,hexahydro-3-(phenylmethyl) (probability of 88.01%).

Conclusion

In conclusion, the isolated bacterium is confirmed to be *Halobacillus *sp.* *and the secondary metabolites produced by this bacterium could be the potential source for the development of novel antimicrobial and antioxidant compounds that are highly biologically compatible. Further research may help to develop novel compounds in the pharmaceutical industry.

## Introduction

Halophilic microorganisms are salt-loving microbes that thrive in higher saline conditions. These microorganisms are mostly prokaryotic and eukaryotic in nature [1,2]. Halophilic microorganisms found in hypersaline conditions are able to endure saline within a certain concentration range. Based on their sodium chloride (NaCl) tolerance, halophilic bacteria were categorized into three classes: slightly, moderately, and extremely halophilic organisms, tolerating 2-5%, 5-20%, and 20-30% of NaCl, respectively [3]. The level of tolerance and salt prerequisites are reliant upon temperature, pH, and development medium. Hence, the survival of halophiles is limited by specific environmental factors. The microorganisms that survive in extreme climates are called polyextremophiles [4,5]. In fact, a halophilic microorganism can likewise be alkaliphile, assigned as haloalkaliphilic, developing ideally or very well at pH values above 9.0, yet it cannot develop at near neutral pH of 6.5 [6]. The general characteristics of halophilic microorganisms include low nutritional need and resistance to a high amount of salt with the ability to adjust the osmotic pressure of the environment [7]. Their mechanism of halo-adaptation depends on a high intracellular concentration of potassium chloride, typically exceeding 37%, or accumulating compatible solutes to regulate the equilibrium of sodium within the cytoplasm and counteract the osmotic pressure of the saline external environment [8]. Halophilic bacteria are potential sources for novel bioactive compounds [9].

Nowadays, antimicrobial drug-resistant pathogens pose a critical danger in medication. Appropriately finding new sources of antimicrobial compounds is crucial for developing effective drugs against these resistant microorganisms [10]. Halophilic microscopic organisms can be an important source of antimicrobial compounds [11]. The secondary metabolites of halophilic microorganisms contain different bioactive mixtures like lipopeptides, polypeptides, polyketides, isocoumarins, and macrolactones [12,13]. Halophilic microorganisms found in salt pans have not been thoroughly investigated as potential sources of antimicrobial compounds, while only a limited number of studies have reported on the antimicrobial capabilities of microorganisms isolated from Indian salt marshes. This study aimed to fill the current knowledge gap by conducting a thorough analysis of the secondary metabolite profile produced by a potent halophilic bacterium and evaluating its associated biological activities.

## Materials and methods

Halophilic bacterium

The Biocontrol and Microbial Product Lab, Department of Microbiology, Saveetha Medical College and Hospital, Thandalam, housed the potent halophilic bacterial isolate stored at 7°C for future use in a nutrient agar slant medium containing 8% NaCl.

Clinical pathogens

Methicillin-resistant *Staphylococcus aureus*, *Enterococcus faecalis*, *Escherichia coli*, *Pseudomonas aeruginosa*, and *Candida* *albicans* were the five pathogenic microscopic organisms used for the antimicrobial assay. The cultures were obtained from the Clinical Microbiology Lab, Department of Microbiology, Saveetha Medical College and Hospital, Thandalam.

Phenotypic and genotypic characteristics of the halophilic isolate

The determination of phenotypic characteristics included morphological, physiological, and biochemical tests for each strain. Colonies on particular media plates were inspected for their characteristics like color and structure; catalase and oxidase tests were carried out for the determination of the bacterial species.

DNA extraction, amplification, and sequencing of 16S rRNA

The 16S rRNA sequencing of potent halophilic bacteria was done in Eurofins Genomics (Bengaluru, India). Briefly, the homogenized single colony was grown in an 8% nutrient broth culture medium and after 24 hours of shaking and the cell-free supernatant was transferred to a 5-mL transport vial and sent for gene sequencing. The DNA was extracted using Qiagen DNeasy kit (Qiagen GmbH, Hilden, Germany). The extracted DNA samples were amplified using universal oligonucleotide primers of 27F (5′-AGAGTTTGATCCTGGCTCAG-3′) and 1492R (5′-TACGGTTACCTTGTTACGACTT-3′) for 16S rRNA gene. The amplified DNA was subjected to electrophoresis on a 0.8% agarose gel and a NanoDrop spectrometer (ND-1000 spectrometer, NanoDrop Technologies, Willington, CT, USA) was used to measure the quality of the genomic DNA [14]. The Sanger sequencing method or chain-termination DNA was used to sequence the amplicons. An automated DNA Analyzer (ABI 3730XL Capillary Sequencers, Applied Biosystems, Bengaluru, India) was used to automate a modified Sanger method commonly used to check the sequence of templates [15].

Bioinformatics

Sequence Scanner v.1.0 (Informer Technologies Inc., Los Angeles, CA, USA) was used to check the quality of the sequence. ChromasPro 1.5 (Informer Technologies Inc.) was used to assemble the sequences after quality checks. The direction of the gathered groupings was checked utilizing OrientationChecker v.1.0 (developed by Ken Ashelford). Using a BLAST 2.0 (basic local alignment search tool) program, bacterial isolates were identified by comparing their DNA sequences to those in the GenBank NCBI (National Center for Biotechnology Information) database. The arrangements were then adjusted by pairwise arrangement utilizing ClustalW, and the phylogenetic tree was built using the neighbor-joining technique [16,17].

Production of microbial metabolites

In a nutrient broth medium containing optimized parameters, the halophilic bacteria were cultivated. Each organism received around 150 mL of the nutrient broth in its own conical flask, and the cultures were shaken and stirred continuously for 72 hours at 35°C. After incubation, the cell-free supernatant was collected by centrifugation. The secondary metabolites were extracted by adding an equivalent volume of ethyl acetate to the supernatant culture in a separating funnel. Ethyl acetate was then extracted (liquid-liquid extraction) from the supernatant. Using a rotary evaporator (Buchi Rotavapor II, Sigma-Aldrich, St. Louis, MO, USA), the resulting extracts were condensed. The samples that included crude metabolites were prepared at a concentration of 2 mg/mL and kept in the refrigerator.

Antimicrobial assay

The well diffusion assay was used to test crude secondary metabolites against five specific pathogens: *S. aureus*, *E. feacalis*, *E*. *coli*, *P. aeruginosa*, and *C. albicans. *Then all the bacterial and fungal pathogens were harvested from the early stationary phase of their growth and concentrations of their cultures were changed to 0.4 O.D. for bacteria and 0.5 O.D. for *C. albicans* using sterile Sabourad Dextrose broth. The grown bacterial pathogens were swabbed on the Mueller-Hinton Agar (MHA). Then using cork, borer wells were made on surface of the agar medium and 100 µL of crude metabolites were loaded into the respective wells. Then the plates were incubated at 37°C for 16 hours. The zone of inhibitions was then measured using a zone scale and results were noted.

Minimum inhibitory concentration (MIC)

The MIC of crude metabolites was determined as per Nandhagopal et al. [18]. In accordance with the CLSI (Clinical and Laboratory Standard Institute) Guidelines, the MIC test was performed on a Tarsons 96-well kit (Tarsons Products Pvt. Ltd., Kolkata, India). All the pathogens were tested in a Muller-Hinton broth (MHB). The first 10 wells on the test plates received various concentrations of crude metabolites (512, 256, 128, 64, 32, 16, 8, 4, 2, and 1 mg/L) while the negative control well received 5 μL of human pathogens at concentrations ranging from 105 to 106 mg/L. For 16 hours at 37°C, the plates were incubated. All the wells were coated with 10 mL of freshly prepared MTT (3-(4,5-dimethylthiazol-2-yl)-2,5-diphenlyltetrazolium bromide) at a concentration of 5 mg/mL following the incubation period. It was then covered with aluminum foil and incubated for 1 hour. A 100 μL of dimethyl sulfoxide (DMSO) was added to the wells as the solubilization arrangement and incubated for 15 to 30 min. Optical density (OD) was recorded at 595 nm on an ELISA reader (Tecan Multimode Reader, Tecan Group Ltd., Männedorf, Switzerland).

Antioxidant activity by DPPH assay

The antioxidant activity was measured using the DPPH (2,2-diphenyl-1-picrylhydrazyl) radical scavenging assay. Briefly, various concentrations (512, 256, 128, 64, 32, 16, 8 and 4 mg/mL) of the sample were prepared in methanol. An equal volume of DPPH solution (0.1 mM in methanol) was added to each sample. The mixture was incubated in the dark for 30 minutes, and the absorbance was measured at 517 nm using a spectrophotometer. The percentage of DPPH radical scavenging activity was calculated using the formula:

$\text{Antioxidant Activity} (\%) = \left( \frac{A_{\text{control}} - A_{\text{sample}}}{A_{\text{control}}} \right) \times 100$

where *A*_control_ is the absorbance of the control and *A*_sample_ is the absorbance of the sample.

Hemolytic activity

To assess the biocompatibility of secondary metabolites derived from the *Halobacillus *sp., experiments were conducted utilizing freshly collected human blood cells. The human blood cells obtained from volunteer participants were washed three times with phosphate-buffered saline (PBS) prior to further analysis. The secondary metabolites were diluted at various concentrations (512, 256, 128, 64, 32, 16, 8 and 4 mg/mL) in 800 μL of PBS solution. A 200 μL of the blood sample was added to each microcentrifuge tube separately. The negative control was PBS, while the positive control was 1% Triton X-100. After the addition of the sample and control, the tubes were incubated for 1 hour at 37°C. The microcentrifuge tubes were centrifuged at 5000 rpm for 7 min. The absorbance value at 570 nm was noted and % hemolysis was calculated as follows:

$\text{Percentage of Hemolysis} (\%) = \left( \frac{A_{\text{sample}} - A_{\text{blank}}}{A_{\text{positive control}} - A_{\text{blank}}} \right) \times 100$

where *A*_sample_ is the absorbance of the sample, *A*_blank_ is the absorbance of the blank, and *A*_positive control_ is the absorbance of the positive sample.

The measurements were done twice and results are presented as mean ± standard deviation (±SD).

Gas chromatography-mass spectrometry (GC-MS) analysis of secondary metabolites

The Trace GC Ultra with Trace DSQ II model mass spectrometer (Thermo Fisher Scientific, Waltham, MA, USA) was utilized for the examination of particles present in the unrefined metabolites. The instrument was set to the injector port temperature of 250°C, connection point temperature of 250°C, source at 200°C, and engine vacuum strain at 40 psi. The oven temperature was customized (70°C for 2 min, 150⁰C at 8°C/min, up to 260°C at 10°C/min). The DB-35 MS GC column (Agilent Technologies, Santa Clara, CA) Non-Polar section was utilized, whose aspects were 0.25mm OD x 0.25 μm ID. At a rate of 1 mL/min, helium served as the carrier gas. The mass spectrometer was set to look for fragments with masses between 50 and 650 Da. The ionization energy was -70 eV, and the mass spectrometer included a pre-filter to remove the neutral particles. The results obtained were compared to the reference data.

Statistical analysis

All tests were done in triplicate and the mean ± standard deviations (SD) was calculated for the acquired data. The chart was plotted utilizing GraphPad Prism 5.0 (Insight Venture Partners LP, New York, USA).

## Results

Morphological and phenotypic characterization of halophilic bacteria

**Figure 1:**
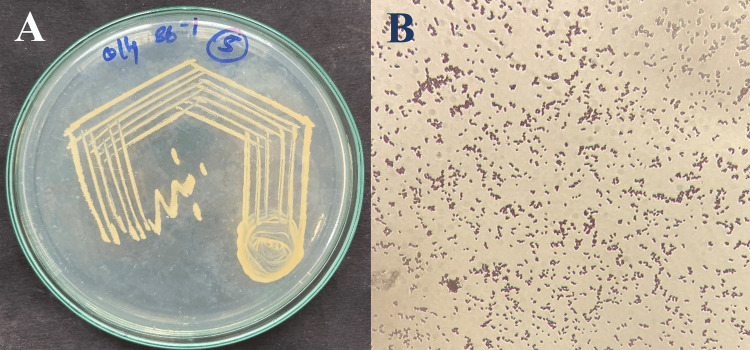
Morphological observation of halophilic bacterium A: Colony morphology; B: Gram's staining

Halophilic bacteria cultured in nutrient agar (NA) medium supplemented with 8% NaCl showed colonies with distinct traits: orange in color, convex shape, and opaque. A subsequent observation using light microscopy (Leica DM1000; Leica Microsystems, Wetzlar, Germany) and Gram's staining revealed the presence of gram-positive bacilli. This suggests the presence of specific halophilic bacteria thriving in the medium (Figure 1).

Different biochemical tests, such as catalase and indole, showed positive results, while methyl red, Voges-Proskauer, citrate utilization, urease, and oxidase tests gave negative results. In the triple sugar iron test, there was only acid production and no CO_2_ and H_2_S was generated.

Molecular identification of bacterial isolates

**Figure 2:**
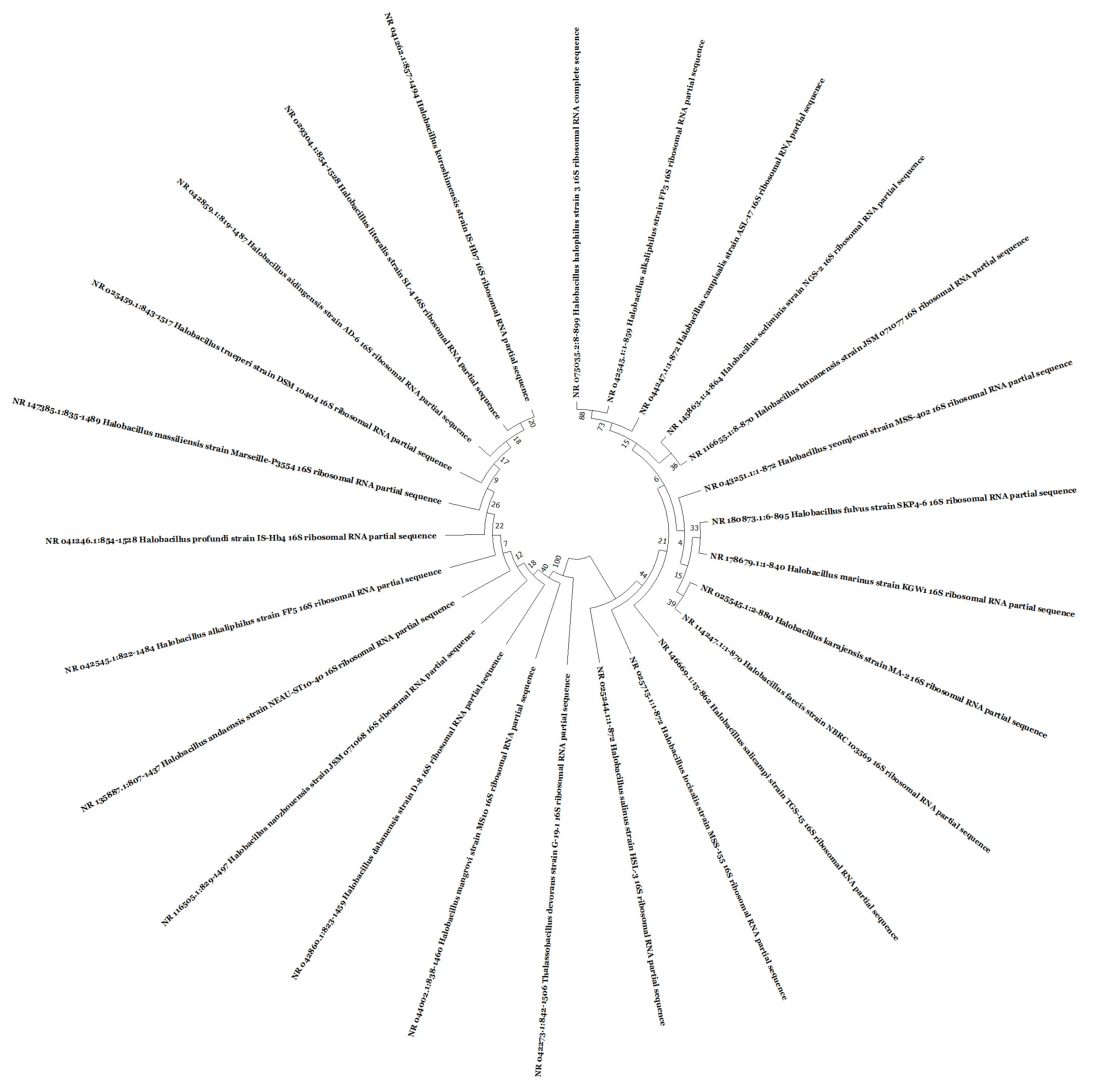
Phylogenetic tree construction for the halophilic bacterium

The DNA of a potent bacterial strain was isolated and subjected to polymerase chain reaction (PCR) for identification through 16S rRNA gene sequencing using the Sanger method. On comparing the DNA sequences in GenBank NCBI database using the BLAST 2.0 program, the results show 99.66% similarity for *Halobacillus* sp. strain PP-M1-14 16S rRNA gene and *Halobacillus* sp. strain PP-HV-20 16S rRNA, 99.65% similarity for *Halobacillus* sp. JSM 077029 16S rRNA and *Halobacillus* sp. SS11-4 16S, 99.54% similarity for *Halobacillus* sp. strain 4TMC2 16S, and 99.53% similarity for *Halobacillus alkaliphilus* strain ZSTB206 16S rRNA, *Halobacillus alkaliphilus* RM-D8 16S rRNA, *Halobacillus *sp. T7-6M gene for 16S rRNA. The *Halobacillus *sp. strain 1ASR15-9 16S showed the maximum similarity with the GenBank NCBI database as shown in Figure 2.

Antimicrobial activity of microbial metabolites

**Figure 3:**
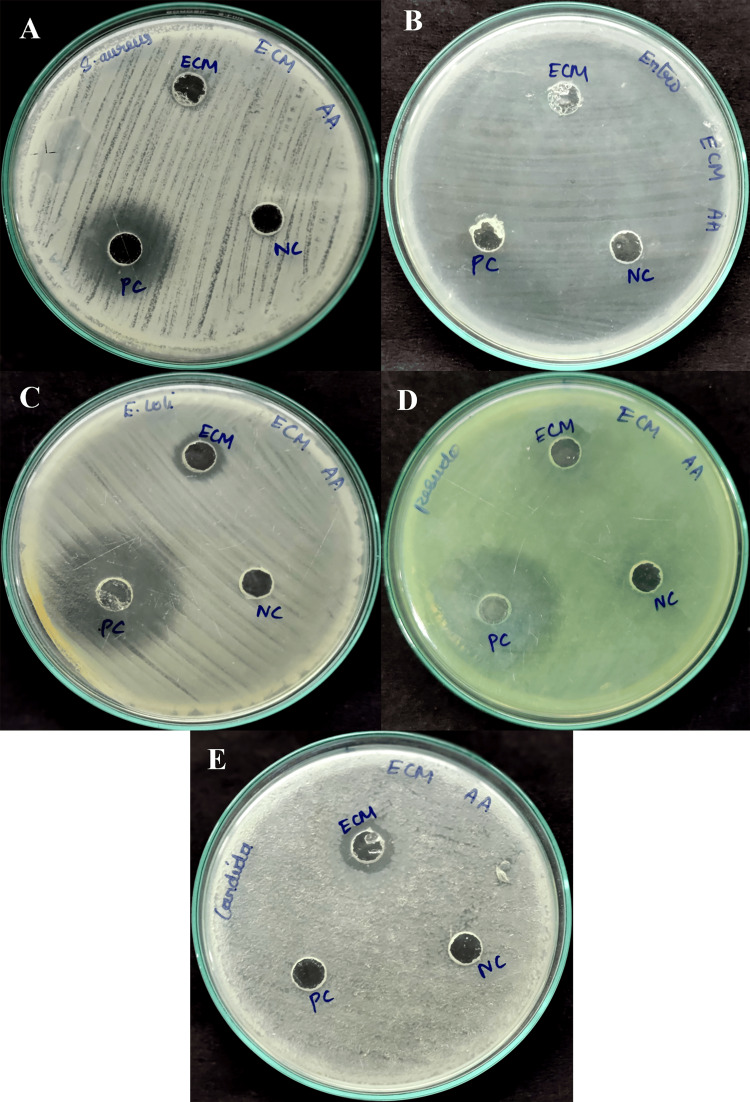
Antimicrobial activity of secondary metabolites extracted from halophilic bacteria A: *S. aureus*; B: *E. faecalis*; C: *E. coli*; D: *P. aeruginosa*; E: *C. albicans*; ECM: extracellular metabolites; PC: positive control; NC: negative control

The study presents the zones of inhibition measured for various pathogens treated with ECM and PC. For Gram-positive bacteria, *S. aureus* showed a zone of inhibition at 11 mm for ECM and 27 mm for PC, while *E. coli *exhibited inhibition at 13 mm for ECM and 29 mm for PC. In the case of Gram-negative bacteria, *P. aeruginosa* had a zone of inhibition at 14 mm for ECM and 32 mm for PC. *C.* *albicans* displayed the zone of inhibition at 13 mm for ECM and no inhibition for PC, whereas *E. faecalis* did not show any inhibition for either treatment (Table 1 and Figure 3).

**Table 1:** Antimicrobial activity of secondary metabolites produced by halophilic bacterium ECM: Extracellular metabolites; PC: positive control

Zone of Inhibition (mm)										
Gram-positive bacterium				Gram-negative bacterium					Fungal pathogen	
S. aureus		E. faecalis		E. coli		P. aeruginosa			C. albicans	
ECM	PC	ECM	PC	ECM	PC	ECM		PC	ECM	PC
											11	27	-	-	13	29	14		32	13	-

Minimum inhibitory concentration

The minimum inhibitory concentration (MIC) values for various pathogens, categorized into fungal pathogens, Gram-negative bacteria, and Gram-positive bacteria, were noted. For the Gram-positive bacterium *S. aureus, *there was no growth inhibition at any of the tested concentrations of ECM, whereas the inhibition of growth was observed at 2 mg/L for PC. *E. faecalis* showed no growth inhibition of ECM but at 512 mg/L for PC. The Gram-negative bacterium *E. coli *exhibited an MIC of 128 mg/L for ECM and 2 mg/L for PC. *P. aeruginosa* showed an MIC of 128 mg/L for ECM and 1 mg/L for PC. Lastly, for the fungal pathogen *C. albicans*, the MIC was 128 mg/L for ECM, but it did not inhibit the growth of PC (Table 2).

**Table 2:** Minimum inhibitory concentrations of secondary crude metabolites ECM: Extracellular metabolites; PC: positive control; ND: not determined

Minimum inhibitory concentration (MIC) (mg/L)									
Gram-positive bacterium				Gram-negative bacterium				Fungal pathogen	
S. aureus		E. faecalis		E. coli		P. aeruginosa		C. albicans	
ECM	PC	ECM	PC	ECM	PC	ECM	PC	ECM	PC
										ND	2	ND	512	128	2	128	1	128	ND

Antioxidant activity of microbial metabolites

**Figure 4:**
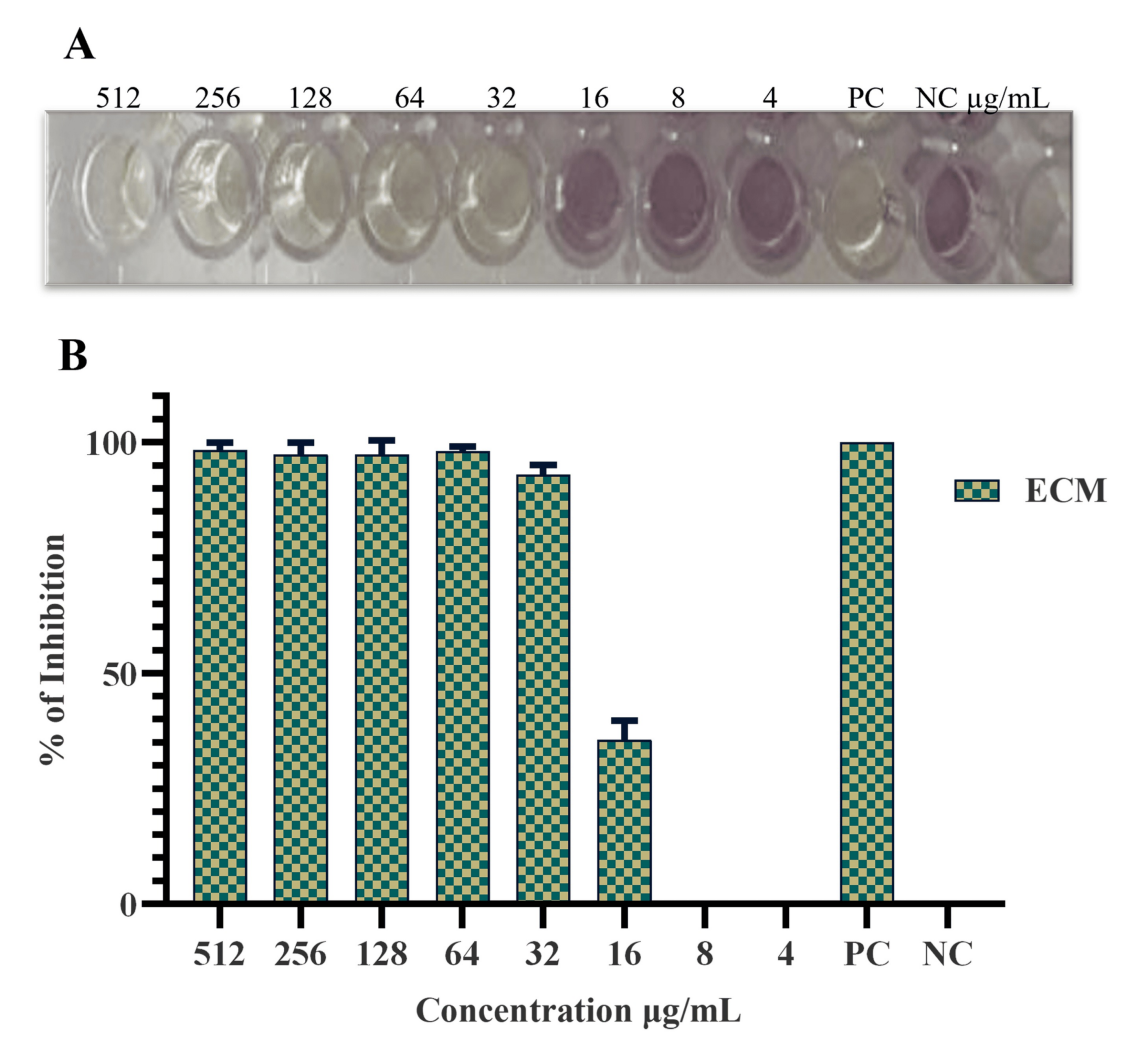
Antioxidant activity of the secondary metabolites of Halobacillus sp. A: Antioxidant activity by DPPH assay; B: Percentage of anti-oxidant activity of ECM. PC: Positive control; NC: negative control; ECM: extracellular metabolites

The antioxidant activity of secondary metabolites from *Halobacillus* sp. was evaluated at various concentrations (in μg/mL). At a concentration of 512 μg/mL, 98.33% antioxidant activity was observed. The activity dropped to 97.33% at 256 μg/mL, but remained consistent at 128 μg/mL. At 64 μg/mL, the antioxidant activity decreased to 96% and further declined to 93% at 32 μg/mL. A significant drop in antioxidant activity was observed at 16 μg/mL, at which it fell to 35.66%. At lower concentrations of 8 and 4 μg/mL, no antioxidant activity was observed. This data illustrates a clear trend of decreasing antioxidant activity as the concentrations of the secondary metabolites decreased (Figure 4A, 4B).

Hemolytic activity

**Figure 5:**
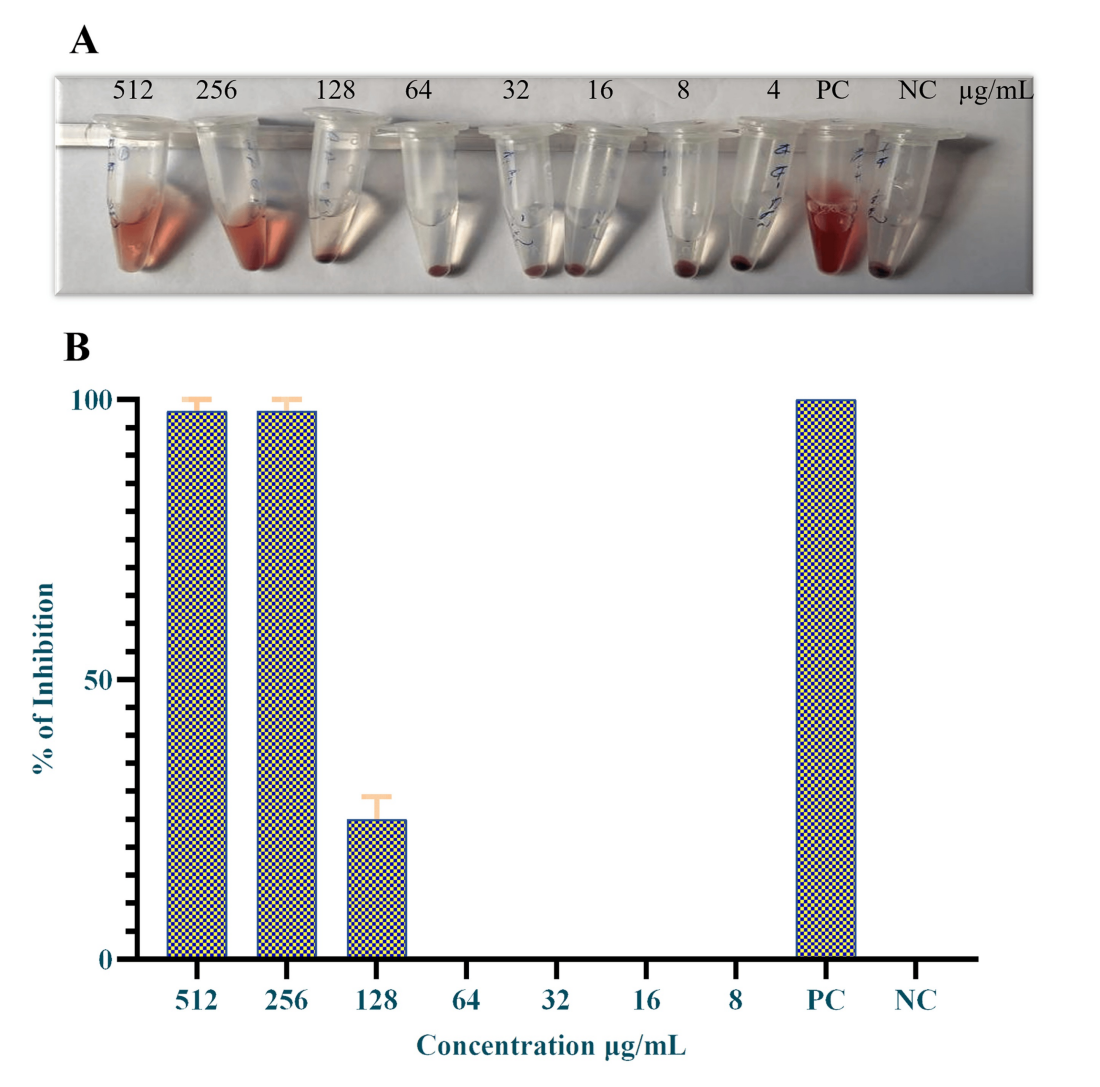
Hemolytic activity of crude metabolites A: Hemolytic activity crude metabolites; B: Percentage of hemolytic activity PC: Positive control; NC: negative control

The study investigated the effects of different concentrations of secondary metabolites produced by *Halobacillus* sp. on red blood cells. The results showed that the secondary metabolites exhibited higher hemolytic activity up to a concentration of 256 µg/mL. At the concentration of 128 µg/mL, the hemolytic activity was moderate (25%), and below 64 µg/mL, no hemolytic activity was observed (Figure 5A, 5B).

GC-MS analysis

**Figure 6:**
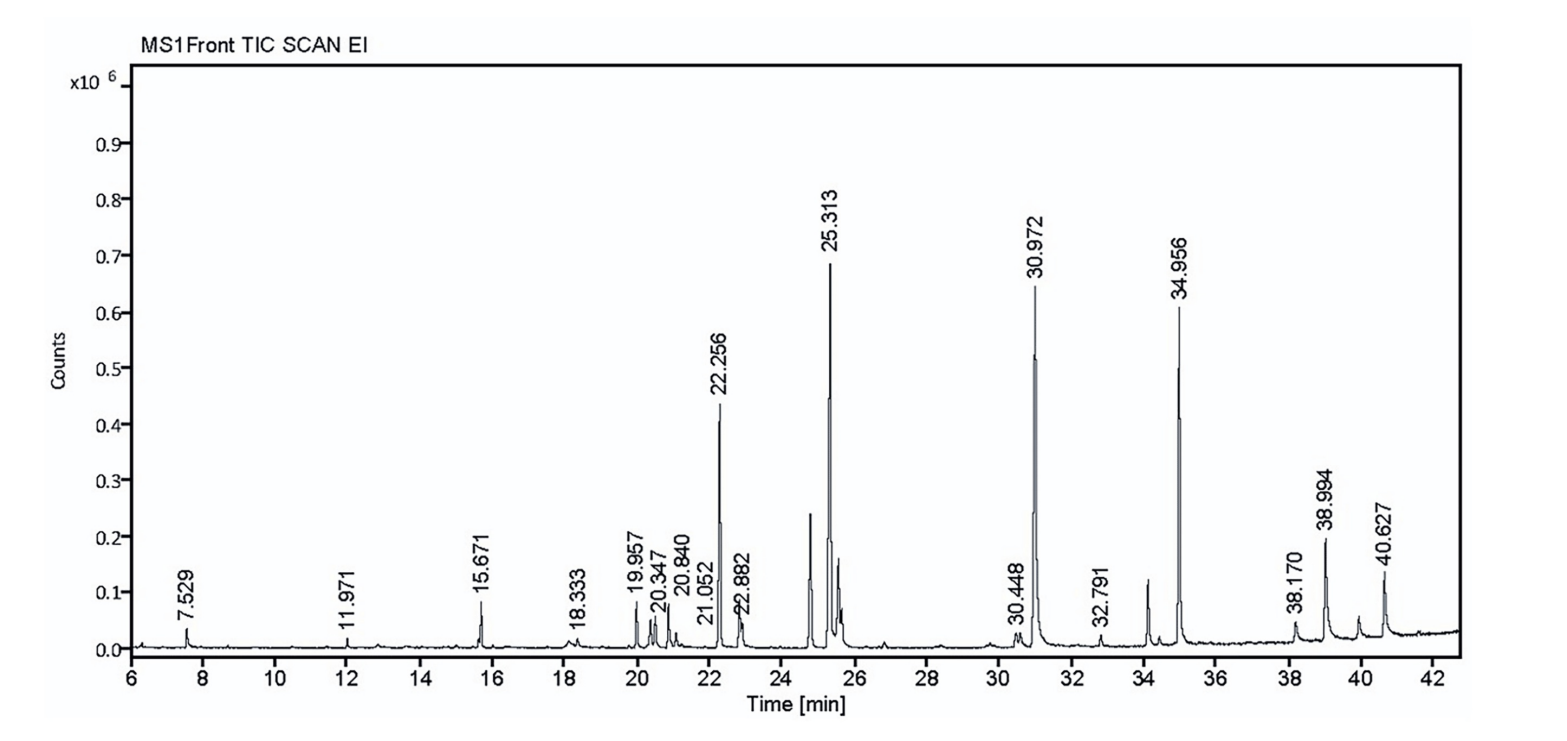
Chromatogram of GC-MS analysis of secondary metabolites

The extracellular crude metabolites were analyzed using GC-MS, and a total of 17 compounds were identified. The major compounds present in the crude metabolites were as follows: cyclo(L-prolyl-L-valine) (probability of 95.63%), pyrrolo[1,2-a]pyrazine- 1,4-dione,hexahydro-3-(2-methylpropl) (probability of 94.45%), pyrrolo[1,2-a]pyrazine-1,4-dione,hexahydro-3-(phenylmethyl)- (probability of 88.01%), and 2,5-piperazinedione,3,6-bis(2-methylpropyl) (probability of 71.94%) (Figure 6 and Table 3).

**Table 3:** GC-MS analysis of crude metabolites extracted from Halobacillus sp.

Peak	R. Time	Area %	Name	Probability %	Formula	Mol. wt
1	7.52	0.98	2-Piperidinone	70	C_6_H_9_NO	99.13
2	11.97	0.36	Phenol, 2-methoxy-3-(2-propenyl)-	21.21	C_10_H_12_O_2_	164.2
3	15.67	1.22	2,4-Di-tert-butylphenol	54.37	C_14_H_22_O	206.32
4	18.33	0.38	2,5-Piperazinedione, 3-methyl-6-(1-methylethyl)-	81.11	C_8_H_14_N_2_O_2_	170.21
5	19.95	2.02	1,4-diazabicyclo[4.3.0]nonan-2,5-dione, 3-methyl	91.95	C_8_H_12_N_2_O_2_	168.19
6	20.34	1.36	DL-Alanyl-l-leucine	72.61	C_9_H_18_N_2_O_3_	202.13
7	20.84	2.21	Pyrrolo[1,2-a]pyrazine-1,4-dione, hexahydro-	74.38	C_7_H_10_N_2_O_2_	154.17
8	21.05	0.8	3-Isobutyl-2,5-piperazinedione	74.44	C_8_H_14_N_2_0_2_	170.21
9	22.256	12.32	Cyclo(L-prolyl-L-valine)	95.63	C_10_H_16_N_2_O_2_	196.25
10	22.88	1.34	3,6-Diisopropylpiperazin-2,5-dione	26.92	C_10_H_18_N_2_O_2_	198.26
11	25.313	23.53	Pyrrolo[1,2-a]pyrazine-1,4-dione, hexahydro-3-(2-methylpropyl)-	94.45	C_11_H_18_N_2_O_2_	210.27
12	30.44	1.1	2,5-Piperazinedione, 3-methyl-6-(phenylmethyl)-	60.66	C_12_H_14_N_2_O_2_	218.15
13	30.97	22.68	2,5-Piperazinedione, 3,6-bis(2-methylpropyl)-	71.94	C_12_H_22_N_2_O_2_	226.17
14	34.95	16.66	Pyrrolo[1,2-a]pyrazine-1,4-dione, hexahydro-3-(phenylmethyl)-	88.01	C_^14^_H_16_N_2_O_2_	244.29
15	38.17	1.24	Pyrimidine-2(1H)-thione, 4,4,6-trimethyl-1-(1-phenylethyl)-	1.24	C_15_H_19_N_2_S	259.39
16	38.994	6.46	Cyclo-(l-leucyl-l-phenylalanyl)	17.67	C_15_H_20_N_2_O_2_	260.33
17	40.62	4.62	Formic acid, (2-fluoro-5-nitrophenyl)methyl ester	19.52	C_8_H_6_FNO_4_	199.14

## Discussion

Halophilic bacteria, which thrive in high-salinity environments, have gained considerable attention due to their potential as a source of novel bioactive compounds. The secondary metabolites, produced by them as part of their adaptation to extreme conditions, possess unique chemical structures and exhibit bioactivities that hold potential for pharmaceutical and biotechnological applications. These secondary metabolites have shown antimicrobial, anticancer, and antioxidant properties, making them valuable for the development of new therapeutics. *Halomonas* is a genus of Gram-negative, moderately halophilic, hydrocarbon-degrading bacteria. They are rod-shaped and are typically found in environments with high salt concentrations, such as salt lakes and saline soils, but they have also been discovered in marine environments [19]. The *Halomonas* spp. are most widely recognized with biotechnological potential in their applications as biosurfactants, emulsifiers, ectoines, and polyhydroxyalkanoates (a type of bioplastic). The *Halomonas* sp. is also used in bioremediation to clean contaminated environments, especially in saline environments, when the pollution is caused by oil. In a study conducted by Kayanadath et al. [19], *Halomonas* sp. (BOB-3), from the Bay of Bengal, produced lipase and a biosurfactant with anti-biofilm properties, which prevented biofilm formation in bacterial pathogens due to the production of various metabolites, which make them potential candidates for clinical and industrial applications [19-21]. However, the halophilic bacteria from salt pans in southern India have not been extensively studied or characterized taxonomically. Additionally, the potential by-products produced by these halophilic bacteria also require further investigation and identification at the species level.

In a study conducted by Joulak et al. [22], the exopolysaccharide (EPS-S6), produced by *Halomonas elongata* S6 consists of mannose, glucosamine, glucose, and rhamnose and has shown different activities. EPS-S6 is extremely thermostable, tolerates temperatures up to 280°C, and also shows anti-biofilm activity against pathogenic strains like *E. coli, S. aureus,* and *E. faecalis*. While comparing EPS-S6 with commercially available butylated hydroxyanisole (BHA), EPS-S6 showed greater radical scavenging activity and provided protection against DNA damage caused by hydroxyl radicals by neutralizing the reactive oxygen species and protecting the DNA [22]. However, in this study, we mainly focused on the antimicrobial and antioxidant properties and biological compatibility of crude metabolites produced by the *Halobacillus* sp.

Similarly, different isolates of *Halomonas* sp. exhibit significant anticancer properties against the HepG2 liver cancer cell line by facilitating cell cycle arrest in the G2/M phase and inducing apoptosis. The production of particular dipeptides and biosurfactants by *Halomonas *strain may be responsible for its anticancer effect. The most potent molecules found were Surfactin C14 and Surfactin C15 [23]. In the present study, the surfuctant-like molecules produced by *Halobacillus* sp. were found to exhibit moderate antimicrobial activity and excellent antioxidant activity. Cyclo(L-prolyl-L-valine), pyrrolo[1,2-a]pyrazine-1,4-dione, hexahydro-3-(2-methylprop), 2,5-piperazinedione,3,6-bis(2-methylpropyl)-, and pyrrolo[1,2-a]pyrazine-1,4-dione,hexahydro-3-(phenylmethyl)- were the major compounds isolated from the metabolites extracted from *Halobacillus* sp. These compounds likely contribute to the maintenance of microbial community structure and function in hypersaline ecosystems. Moreover, the study of halophilic bacteria and their secondary metabolites can provide insights into the mechanisms of adaptation to high-salinity environments. Understanding these mechanisms can help in the development of strategies to overcome salinity-related challenges in various industries, such as agriculture and bioremediation. Overall, the importance of halophilic bacteria and their secondary metabolites lies in their potential as a source of novel bioactive compounds that have pharmaceutical applications.

Limitations of the study

The species of the potent halophilic bacterium has to be classified and the optimization of culture conditions for specific organisms is required. While bacteria produce a vast array of bioactive molecules, identifying those with potential therapeutic properties will be challenging. Identifying bioactive molecules from natural sources, finding their mode of action, and the study of their pharmacokinetics in living organisms are time-consuming.

## Conclusions

In conclusion, the secondary metabolites produced by the potent bacterium *Halobacillus* sp. show promising antimicrobial activity against various pathogens and demonstrate excellent antioxidant properties. They have potential applications in clinical settings in which the halophilic bacteria offer an exciting opportunity for discovering new biomolecules with potential benefits in medicine, industry, and environmental management. Further research is essential to fully utilize their unique capabilities.
